# The effects of psychiatric disorders on the risk of chronic heart failure: a univariable and multivariable Mendelian randomization study

**DOI:** 10.3389/fpubh.2024.1306150

**Published:** 2024-01-17

**Authors:** Yang Chen, Wenke Peng, Min Pang, Botao Zhu, Huixing Liu, Die Hu, Yonghong Luo, Shuai Wang, Sha Wu, Jia He, Yang Yang, Daoquan Peng

**Affiliations:** ^1^Department of Cardiovascular Medicine, The Second Xiangya Hospital, Central South University, Changsha, China; ^2^Research Institute of Blood Lipid and Atherosclerosis, Central South University, Changsha, China

**Keywords:** chronic heart failure, psychological disorders, univariable Mendelian, multivariable Mendelian randomization, causal relationship

## Abstract

**Background:**

Substantial evidence suggests an association between psychiatric disorders and chronic heart failure. However, further investigation is needed to confirm the causal relationship between these psychiatric disorders and chronic heart failure. To address this, we evaluated the potential effects of five psychiatric disorders on chronic heart failure using two-sample Mendelian Randomization (MR).

**Methods:**

We selected single nucleotide polymorphisms (SNPs) associated with chronic heart failure and five psychiatric disorders (Attention-Deficit Hyperactivity Disorder (ADHD), Autism Spectrum Disorder (ASD), Major Depression, Bipolar Disorder and Schizophrenia (SCZ)). Univariable (UVMR) and multivariable two-sample Mendelian Randomization (MVMR) were employed to assess causality between these conditions. Ever smoked and alcohol consumption were controlled for mediating effects in the multivariable MR. The inverse variance weighting (IVW) and Wald ratio estimator methods served as the primary analytical methods for estimating potential causal effects. MR-Egger and weighted median analyses were also conducted to validate the results. Sensitivity analyses included the funnel plot, leave-one-out, and MR-Egger intercept tests. Additionally, potential mediators were investigated through risk factor analyses.

**Results:**

Genetically predicted heart failure was significantly associated with ADHD (odds ratio (OR), 1.12; 95% CI, 1.04–1.20; *p* = 0.001), ASD (OR, 1.29; 95% CI, 1.07–1.56; *p* = 0.008), bipolar disorder (OR, 0.89; 95% CI, 0.83–0.96; *p* = 0.001), major depression (OR, 1.15; 95% CI, 1.03–1.29; *p* = 0.015), SCZ (OR, 1.04; 95% CI, 1.00–1.07; *p* = 0.024). Several risk factors for heart failure are implicated in the above cause-and-effect relationship, including ever smoked and alcohol consumption.

**Conclusion:**

Our study demonstrated ADHD, ASD, SCZ and major depression may have a causal relationship with an increased risk of heart failure. In contrast, bipolar disorder was associated with a reduced risk of heart failure, which could potentially be mediated by ever smoked and alcohol consumption. Therefore, prevention strategies for heart failure should also incorporate mental health considerations, and vice versa.

## Introduction

Chronic heart failure (HF) is a rapidly growing public health problem with an estimated prevalence of more than 37.7 million people worldwide (1). Psychiatric disorders, such as depression, also constitute a substantial burden on public health (2). Notably, there is a high prevalence of psychological disorders, including depression, in patients with cardiovascular diseases, particularly heart failure (3). Previous studies (4–6) demonstrated significant associations between psychiatric conditions like schizophrenia, bipolar mood disorder, and depression, and increased 30-day and overall readmission rates among African-American heart failure patients. Furthermore, heart failure patients with severe psychiatric disorders have been found to experience adverse outcomes and higher postprocedural mortality rates. These findings suggest a link between heart failure and psychological issues; however, determining the causal relationship between them is crucial for guiding treatment strategies in clinical settings.

Mendelian randomization (MR) is a method that utilizes genetic variants, specifically single nucleotide polymorphisms (SNPs), as instrumental variables (IVs) to establish causal relationships between diseases (outcomes) and risk factors (exposures) (7). The research design of MR adheres to the Mendelian inheritance principle, wherein parents’ alleles are randomly assigned to their offspring, and the natural causal effects of genetic variants on phenotypes are observed. SNPs are independent of potential confounding factors and strongly related to exposure factors (8). Mendelian randomization relies on three assumptions: (1) instrumental variables and exposure factors are strongly correlated; (2) instrumental variables and confounders are not correlated; and (3) instrumental variables are not directly correlated with the outcome, and their effect on the outcome can only be manifested through exposure (9). Two-sample Mendelian randomization (2SMR) is a technique for estimating the causal effect of exposure on outcomes using genome-wide association study (GWAS) summary data (10). To date, two-sample Mendelian randomization has been widely employed in the field of cardiovascular diseases, including heart failure (11–13). However, the causal relationship between psychiatric disorders and heart failure remains largely unexplored. Therefore, we conducted a two-sample MR analysis using summary data from the GWAS to assess the causal effect of five psychiatric disorders (i.e., ADHD, ASD, SCZ, bipolar disorder and major depression) on the risk of heart failure.

Consequently, we carried out a two-sample MR analysis using summary data from GWAS to evaluate the possible causal impact of five psychiatric disorders—namely, attention deficit hyperactivity disorder (ADHD), autism spectrum disorder (ASD), schizophrenia (SCZ), bipolar disorder and major depression—on the risk of heart failure.

## Method

### Study design

**Figure 1:**
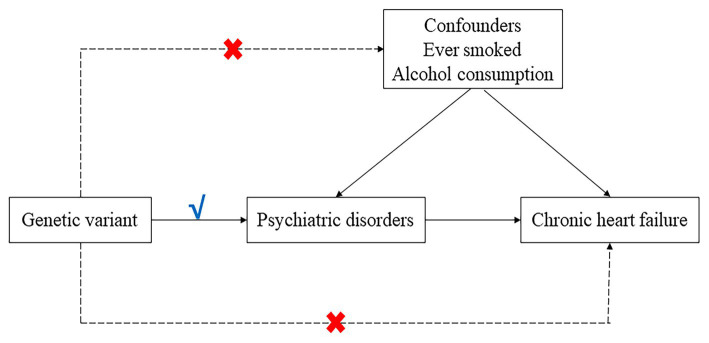
Directed a cyclic graph model of the causal effect between psychiatric disorders and heart failure.

An overview of the two-sample Mendelian randomization (MR) analysis conducted in this study is provided in Figure 1. We employed both univariable and multivariable Mendelian randomization (UVMR and MVMR) using single nucleotide polymorphisms (SNPs) as instrumental variables for the psychiatric disorders to assess the causality between five psychiatric disorders (ADHD, ASD, SCZ, bipolar disorder and major depression) and heart failure. Additionally, we analyzed several potential mediating factors, such as ever smoked and alcohol consumption, to explore the genetic mechanisms of psychiatric disorders and heart failure independent of potential confounders. The study was conducted in accordance with the Enhancing the Quality and Transparency of Health Research (EQUATOR) guidelines (7). All MR analyses satisfied the three basic assumptions: (1) instrumental variables and exposure factors are strongly correlated; (2) instrumental variables and confounders are not correlated; and (3) instrumental variables are not directly correlated with the outcome, and their effect on the outcome can only be reflected through exposure.

### Data sources

A detailed overview of all data sources is provided in Table 1. We obtained publicly available summary statistics from genome-wide association studies (GWAS) to conduct the MR analyses. The summary statistics for the outcome of heart failure included 47,309 cases and 930,014 controls, from 26 cohorts (with a total of 29 distinct datasets). Cases consisted of participants with a clinical diagnosis of heart failure of any etiology, without inclusion criteria based on left ventricular (LV) ejection fraction; controls were participants without heart failure (14).

**Table 1:** Overview of data sources of this Mendelian randomization study on psychiatric disorders and heart failure.

Consortium	Participants	Phenotype	Population	Type of variables	PMID and/or web link
PCG	20,183 cases and 35,191 controls	ADHD	European	Exposure	30478444
iPSYCH-PGC	18,382 cases and 27,969 controls	ASD	European	Exposure	30804558
PGC	170,756 cases and 329,443 controls	Major depression	European	Exposure	30718901
Bipolar Disorder Working Group of the PGC	20,352 cases and 31,358 controls	Bipolar disorder	European	Exposure	31043756
Within family GWAS consortium	47,517 individuals	Depressive symptoms	European	Exposure	35534559
Schizophrenia Working Group of the PGC	33,640 cases and 43,456 controls	Schizophrenia(SCZ)	European	Exposure	25056061
Within family GWAS consortium	99,996 individuals	Ever smoked	European	Confounder	35534559
Within family GWAS consortium	83,626 individuals	Alcohol consumption	European	Confounder	35534559
GIANT	681,275 individuals	body mass index	European	Confounder	30124842
NA	47,309 cases and 930,014 controls	Heart Failure	European	Outcome	31919418

For all psychiatric disorders, GWAS datasets obtained in our study included: ADHD with 20,183 cases and 35,191 controls from 12 cohorts from Psychiatric Genomics Consortium (PGC) (15); ASD with 18,382 cases and 27,969 controls from the iPSYCH-PGC (16); bipolar disorder with 20,352 cases and 31,358 controls from Bipolar Disorder Working Group of the Psychiatric Genomics Consortium (17); major depression with 170,756 cases and 329,443 controls from the PGC (18); SCZ with 33,640 cases and 43,456 controls subjects from the Schizophrenia Working Group of the Psychiatric Genomics Consortium (19); ever smoked with 99,996 subjects from Within family GWAS consortium (20); alcohol consumption with 83,626 subjects from Within family GWAS consortium (20). GWAS datasets of two potential confounders obtained were from within family GWAS consortium. All participants of the original studies provided written informed consent.

Exposed genetic instrumental variables (IVs) were selected at a genome-wide significance level (*p* < 5 × 10^^-8^). Then we used the PLINK algorithm to exclude SNPs from the linkage disequilibrium within a region of 5,000 Kb, with linkage disequilibrium not exceeding the limited r^^2^ value of 0.001(except for SCZ, we set the threshold at r^^2^ value of 0.01) and not being palindromic with intermediate allele frequencies. According to previous studies, the exposure-related F statistic of the instrument is significantly higher than 10 (21) (Supplementary material). Additionally, two-sample MR assumed independence between exposure data and outcome data. Therefore, data on psychiatric disorders with significant overlapping cohorts with heart failure were excluded.

### Statistical analysis

The MR study was conducted in R version 4.1.2 (R Development Core Team, Vienna, Austria) using the “Two-Sample MR” R package version 0.5.6. Two-sample MR and multivariate MR analyses were conducted by the functions mr and mv_multiple, respectively (22). The multiplicative inverse variance-weighted (IVW) method was used in the univariable MR analysis (23). IVW was as the primary analysis to estimate the associations between psychiatric disorders and chronic heart failure. Heterogeneity test, pleiotropy test (MR-Egger intercept test), and leave-one-out analysis were used to estimate sensitivity analysis. For multivariable analysis, we included ever-smoked status and alcohol consumption in the analysis for adjustment. The IVW method was also used for the causal estimates in the multivariable analysis.

## Results

### Two-sample Mendelian randomization of psychiatric disorders (exposure) on heart failure (outcome)

**Figure 2:**
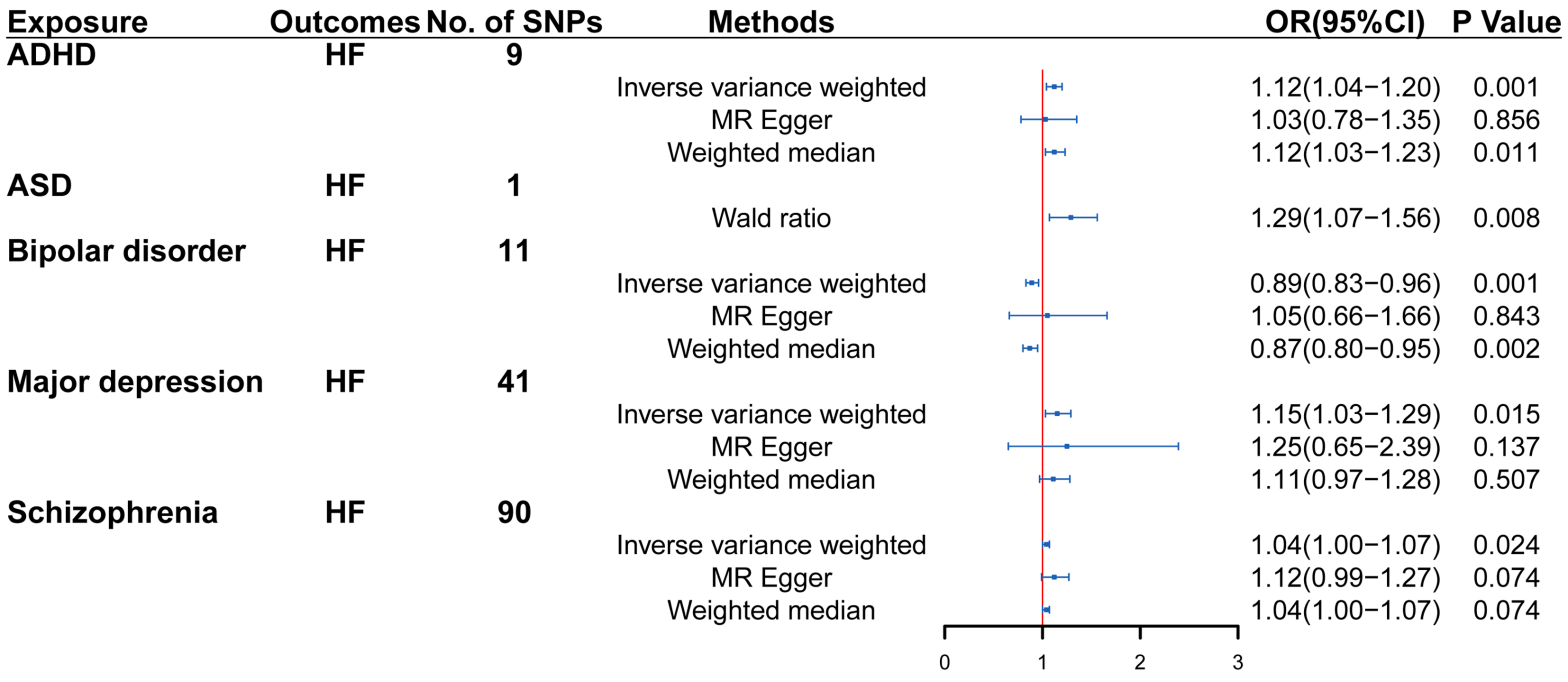
Mendelian randomization (MR) association between genetically predicted psychiatric disorders and heart failure. Odds ratios (ORs) are scaled to the risk of heart failure for genetically predicted psychiatric disorders. HF, heart failure; CI, confidence interval.

Univariable MR analysis suggested that there were 9, 1, 11, 41, and 90 IVs for ADHD, ASD, bipolar disorder, major depression and SCZ, respectively. Genetically determined ADHD, ASD, major depression, bipolar disorder and SCZ exhibited a possible causal effect on heart failure (ADHD: OR = 1.12, 95% CI = 1.04–1.20, *p* = 0.001; ASD: OR = 1.29, 95% CI = 1.07–1.56, *p* = 0.008; major depression: OR = 1.15, 95% CI = 1.03–1.29, *p* = 0.015; SCZ: OR = 1.04, 95% CI = 1.00–1.07, *p* = 0.024; bipolar disorder: OR = 0.89, 95% CI = 0.83–0.96, *p* = 0.001). The causal estimated effect of psychiatric disorders was broadly consistent with heart failure. The effect estimator for the five psychiatric disorders was robust across IVW; however, it was not consistent across weighted median and MR Egger (Figure 2).

### Multivariable Mendelian randomization psychiatric disorders (exposure) on heart failure (outcome)

**Figure 3:**
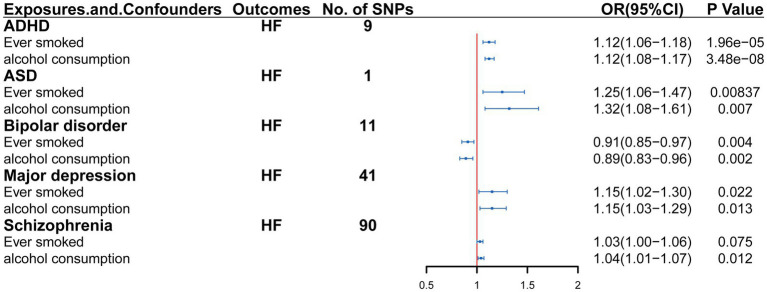
Multivariable Mendelian randomization analysis of the effect of psychiatric disorders on heart failure. OR, Odds ratio; CI, confidence interval.

In multivariable MR analysis adjusting ever-smoked and alcohol consumption, there are strong evidence that ADHD, ASD, bipolar disorder, major depression and SCZ may have possible direct causal effect on heart failure risk (controlling for ever smoked: IVW: OR = 1.12, 95%CI = 1.06–1.18, *p* = 1.96E-05; controlling for alcohol consumption: IVW: OR = 1.12, 95%CI = 1.08–1.17, *p* = 3.48E-08; controlling for ever smoked: IVW: OR = 1.26, 95%CI = 1.06–1.47, *p* = 8.37E-03; controlling for alcohol consumption: IVW: OR = 1.32, 95%CI = 1.08–1.61, *p* = 0.007; controlling for ever smoked: IVW: OR = 0.91, 95%CI = 0.85–0.97, *p* = 0.004; controlling for alcohol consumption: IVW: OR = 0.89, 95%CI = 0.83–0.96, *p* = 0.002; controlling for ever smoked: IVW: OR = 1.15, 95%CI = 1.02–1.30, *p* = 0.022; controlling for alcohol consumption: IVW: OR = 1.15, 95%CI = 1.03–1.29, *p* = 0.013; controlling for ever smoked: IVW: OR = 1.03, 95%CI = 1.00–1.06, *p* = 0.075; controlling for alcohol consumption: IVW: OR = 1.04, 95%CI = 1.01–1.07, *p* = 0.012) (Figure 3).

## Discussion

This is the first MR study to reveal the potential possible causal relationship among the ADHD, ASD, SCZ, bipolar disorder and major depression and heart failure based on large GWAS summary-level data. We discovered genetic evidence that ADHD, ASD, SCZ and major depression were associated with an increased risk of heart failure. Impressively, these adverse possible causal effects remained robust in both univariate and multivariate MR analyses. Other Mendelian randomization study (24) have shown that ASD is associated with a higher risk of heart failure, which is consistent with the findings of our study (though the study did not report on the other psychiatric disorders). Interestingly, our study revealed that bipolar disorder was associated with a decreased risk of heart failure.

The primary objective of multivariate MR model was to assess the effect of multiple relevant exposures on outcomes. Under a range of underlying scenarios, a secondary exposure acts variously as a confounder, a mediator, a pleiotropic pathway and a collider (25). The most significant advantage of MVMR analysis is the robust estimation of the direct effect of each exposure on the outcome in all the scenarios mentioned above. Notably, increased genetic liability to ADHD, ASD, SCZ and major depression was associated with heart failure risk after adjusting for the effects of ADHD, ASD, SCZ and major depression on ever smoked and alcohol consumption, both separately and in a combined model considering potential confounders. However, a decreased genetic liability to bipolar disorder was associated with a reduced risk of heart failure after adjusting for the effects of bipolar disorder on ever smoked and alcohol consumption separately. Although the relationship between bipolar disorder and heart failure presents contrasting results to those reported (26–29), it does not necessarily indicate an issue with our findings. In cases where Mendelian randomization studies themselves have a large number of gene–environment interactions, the influence of genes on disease may be influenced by environmental factors that vary over time or between populations (30). This phenomenon warrants further in-depth exploration.

Furthermore, we conducted a study to systematically assess the causal impact of attention deficit hyperactivity disorder (ADHD), autism spectrum disorder (ASD), schizophrenia (SCZ), bipolar disorder and major depressive disorder and heart failure using two sample Mendelian randomization and multivariable Mendelian randomization (MVMR) analyses, including sensitivity analysis. Additionally, we evaluated the effect of confounding factors such as ever smoked and alcohol consumption on the impact of psychological disorders on heart failure. A Mendelian randomization study (31) on the impact of smoking on heart failure revealed a genetic liability to long-term smoking and a higher lifetime smoking burden associated with a higher risk of HF. Other studies (32, 33) have shown that alcohol consumption increases the risk of cardiovascular disease. Biddinger et al. (34) assessed the association of habitual alcohol intake with cardiovascular disease risk. Using multivariate Mendelian randomization analysis of ever smoked, alcohol consumption, psychological disorders (exposure), and heart failure (outcome), we found a strong correlation between psychological disorders and heart failure, even when correcting for confounding factors such as ever smoked and alcohol consumption. These results further suggest that psychological disorders increase the risk of heart failure (except for bipolar disorder). Our results were largely robust to several sensitivity analyses, and by consensus, the Mendelian randomization results on psychological disorders and heart failure are plausible, with psychological disorders increasing the risk of heart failure.

Heart failure is common in adults and accounts for a substantial morbidity and mortality all in the world (35). Furthermore, the pathophysiological process of heart failure can be complicated. Recent studies (36) have shown that activation of the inflammatory response, oxidative stress (37), mitochondrial dysfunction (38), cardiometabolic functional abnormalities (39), myocardial fibrosis (40), endothelial disfunction (41) contribute to cardiac remodeling, leading to an exacerbation of heart failure. We have found that psychological disorders such as depression and anxiety can affect heart patients (42). The underlying mechanisms of psychological disorders in the development of heart failure are not fully understood. Psychological disorders can cause hypercortisolism as well as decreased response to pro-adrenocorticotropic hormone releasing factor (43), activation of platelet function (44), acute stress (45), cardiac arrhythmias (46), endothelial dysfunction (47, 48), activation of mechanisms leading to atherosclerosis, increased inflammatory response (49, 50), etc. There are several possible ways to explain the casual role of psychological disorders in the etiology of heart failure. Psychological disorders appear to promote the secretion of several proinflammatory cytokines, such as CRP and TNF-α (49), affects the level of hormones and vascular endothelium and heart rate. The Mendelian randomization study of psychological disorders and heart failure also revealed that psychological disorders increase the risk of heart failure and may provide a clinical basis for it. All of these psychiatric disorders caused by pathophysiological factors are more or less involved in the progression of heart failure. While the impact of comorbid psychological disorders on heart failure (HF) morbidity and mortality is well recognized, addressing these issues as a routine part of clinical practice holds the potential to enhance quality of life, reduce hospitalizations, improve the cost-effectiveness of care, and positively influence cardiovascular outcomes in patients with HF (42).

The present MR study elucidates the association between psychological disorders and heart failure. This MR study elucidates several merits of the association between genetic liability for psychological disorders and heart failure. The main strengths include the main advantages include the MR design, which mitigates confounding factor relationships, and another advantage is the restriction of the population to European ancestry minimizes bias due to racial heterogeneity. Nevertheless, there are some shortcomings that need to be noted. First, Most of the exposures and outcomes used in our study are binary, and therefore, the Wald-type estimators may bias the causal OR (51). Second, patients with psychiatric disorders may be taking psychotropic drugs, which may have an effect on heart failure, which our study did not analyze. This could also be a direction for future research to support the influence of psychiatric disorders on heart failure. Third, In addition to ever smoked and alcohol consumption as confounders, there may be other confounders that require further exploration in the future. Due to the large sample size of the MR analysis, we believe that the estimated effect will be close to the real situation. However, the homogeneity of the population may limit the generalizability of the results to other populations. Other limitation of this paper, our results demonstrate that the relationship between bipolar disorder and heart failure exhibits the opposite results to those reported, a phenomenon that currently deserves further exploration. It may be that the influence of genes on disease may be influenced by environmental factors that vary from time to time or between populations.

In conclusion, our findings contribute to the growing evidence surrounding the adverse effects of psychological disorders (ADHD, autism spectrum disorder (ASD), schizophrenia (SCZ) and major depressive disorder) on heart failure risk. This highlights the importance of improved diagnosis and management of psychiatric disorders. We believe that in the future, large RCT for psychiatric disorders and heart failure can be conducted, and the population can be expanded to other continents; alternatively, differential SNPs can be concretized into intermediate phenotypes, which can increase the target of clinical intervention.

## Data availability statement

The datasets presented in this study can be found in online repositories. The names of the repository/repositories and accession number(s) can be found at: https://gwas.mrcieu.ac.uk/.

## Ethics statement

Ethical approval was not required for the studies involving humans because we obtained the database from GWSA for analysis. The studies were conducted in accordance with the local legislation and institutional requirements. We don't have an ethics committee waived the requirement of written informed consent for participation from the participants or the participants’ legal guardians/next of kin because We obtained the database from GWSA for analysis.

## Author contributions

YC: Investigation, Methodology, Validation, Writing – original draft, Writing – review & editing. WP: Investigation, Methodology, Validation, Writing – review & editing. MP: Investigation, Methodology, Validation, Writing – review & editing. BZ: Investigation, Methodology, Validation, Writing – review & editing. HL: Investigation, Methodology, Validation, Writing – review & editing. DH: Conceptualization, Investigation, Methodology, Software, Writing – review & editing. YL: Conceptualization, Investigation, Methodology, Software, Writing – review & editing. SWa: Conceptualization, Investigation, Methodology, Software, Writing – review & editing. SWu: Conceptualization, Data curation, Investigation, Validation, Writing – review & editing. JH: Conceptualization, Data curation, Investigation, Validation, Writing – review & editing. YY: Conceptualization, Data curation, Investigation, Methodology, Validation, Writing – review & editing. DP: Project administration, Supervision, Writing – review & editing.
